# Incidental treatment of granulomatosis with polyangiitis with a Janus Kinase inhibitor in a patient undergoing alopecia treatment

**DOI:** 10.1016/j.jdcr.2025.06.046

**Published:** 2025-07-22

**Authors:** Daniela Mendez, Laura Xiang, Wilma Bergfeld

**Affiliations:** Department of Dermatology, Cleveland Clinic Foundation, Cleveland, Ohio

**Keywords:** alopecia, alopecia areata, baricitinib, GPA, granulomatosis with polyangiitis, JAK inhibitors

## Introduction

Granulomatosis with polyangiitis (GPA), a rare systemic vasculitis, primarily affects the respiratory tract and kidneys and is treated with corticosteroids and immunosuppressive agents, including methotrexate, rituximab, and cyclophosphamide. However, long-term use of these therapies poses significant risks such as infections, osteoporosis, and Cushing’s syndrome. Janus kinase (JAK) inhibitors, such as baricitinib, are emerging treatments for autoimmune diseases like rheumatoid arthritis and alopecia areata, although their use in vasculitis remains largely unstudied. JAK inhibitors work by targeting the JAK family of intracellular tyrosine kinases, which are critical components of the JAK-signal transducers and activators of transcription signaling pathway. This pathway is essential for the transmission of signals from various cytokine receptors to the cell nucleus, influencing gene expression and regulating immune responses, cell growth, and hematopoiesis. By inhibiting JAK activity, these inhibitors prevent the phosphorylation and activation of signal transducers and activators of transcription proteins, thereby disrupting downstream signaling and reducing the expression of proinflammatory cytokines and other mediators involved in autoimmune and inflammatory diseases.^1^, ^2^, ^3^ This case report presents a 69-year-old female with refractory GPA, who, despite intolerance to conventional therapies, achieved unexpected remission of GPA while being treated with baricitinib for alopecia areata universalis.

## Case presentation

A 69-year-old female with a history of GPA, diagnosed in 2013, presented to rheumatology with chronic cough, sinusitis, and a positive cytoplasmic antineutrophil cytoplasmic antibodies. She initially received treatment with prednisone and methotrexate, but the latter was discontinued because of mouth sores. She was then treated with rituximab but stopped because of adverse reactions, including ear pain, headaches, and insomnia. Subsequent treatments with azathioprine, leflunomide, methotrexate, and abatacept were either ineffective or poorly tolerated. By 2021, she remained on prednisone, which led to drug-induced Cushing’s syndrome and osteopenia. The patient unfortunately experienced several flares of GPA associated with elevated creatinine and symptoms including chronic cough, shortness of breath, tinnitus, and eye discomfort.

In addition to GPA, the patient has a complex medical history of alopecia, polyarticular psoriatic arthritis, hypertension, thyroid nodule, and stage 3b chronic kidney disease. The alopecia areata universalis has been resistant to previous treatments such as oral and topical minoxidil, intralesional triamcinolone, and topical clobetasol. In August 2022, baricitinib 2 mg was initiated for the alopecia, and prednisone 1 mg was continued for the GPA. Over the next year, the baricitinib dose was increased to 4 mg, whereas prednisone was gradually tapered and completely discontinued by October 2024.

**Fig 1:**
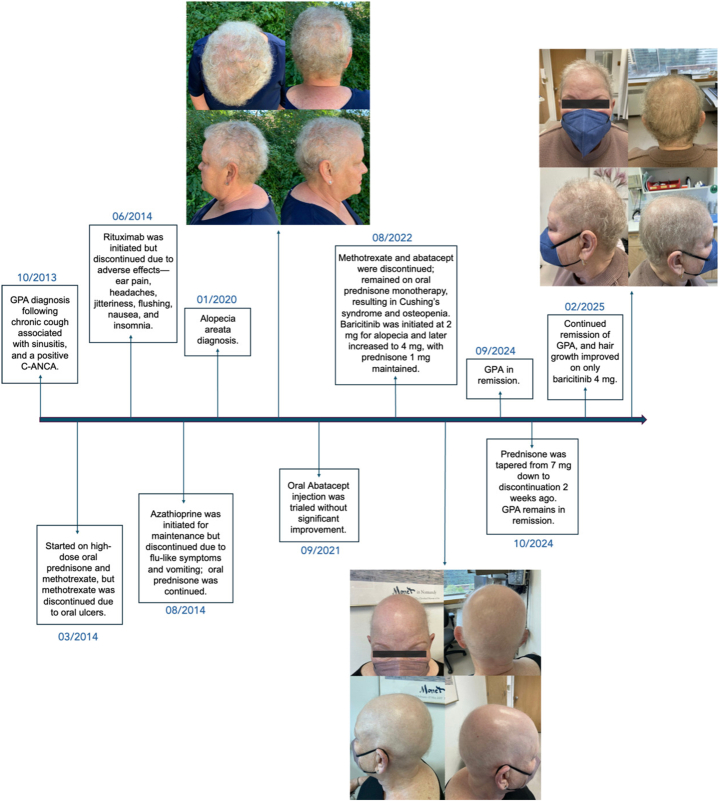
Timeline of diagnosis, treatment, and hair regrowth.

Remarkably, the GPA remained in remission with the use of only baricitinib after tapering off prednisone in October 2024. The stable condition is further supported by laboratory values (creatinine, C-reactive protein, and erythrocyte sedimentation rate) and lack of symptoms or signs of active disease. The psoriatic arthritis symptoms also showed significant improvement. For the alopecia, after starting baricitinib, the patient began developing wispy hairs and fine brown and white fuzz on the scalp (Fig 1). Since being on therapy for over 1 year, she has had significant scalp regrowth, with short baby hair and a mix of fine white and brown strands (Fig 1).

## Discussion

JAK inhibitors have demonstrated therapeutic potential beyond their US Food and Drug Administration-approved indications, showing efficacy across a broad spectrum of inflammatory and immune-mediated conditions. Initially developed for rheumatoid arthritis and select dermatologic diseases, emerging evidence supports their role in treating autoimmune and inflammatory disorders such as inflammatory bowel disease, systemic lupus erythematosus, alopecia areata, vasculitis, and patients with coexisting inflammatory and autoimmune diseases. This case suggests a promising role for JAK inhibitors in the management of refractory GPA and overlapping autoimmune diseases.

Although data on JAK inhibitors for GPA are limited, 2 case series highlight their potential efficacy.^4^^,^^5^ One study of 10 patients with antineutrophil cytoplasm antibody-associated vasculitis, including 6 with GPA, found that tofacitinib induced complete remission in 9 patients and partial remission in 1, with no relapses or significant safety concerns over a median follow-up of 9.5 months.^4^ Another case series evaluated the use of tofacitinib in 11 patients with refractory GPA, and their findings suggested that tofacitinib may be a viable, glucocorticoid-sparing option for managing refractory GPA with granulomatous manifestations.^5^ These results align with our case report.

Some studies have demonstrated the efficacy of JAK inhibitors in large-vessel vasculitis, including giant cell arteritis and Takayasu arteritis.^6^^,^^7^ A retrospective case series reported that in giant cell arteritis, baricitinib and tofacitinib reduced inflammatory markers (C-reactive protein and erythrocyte sedimentation rate) and corticosteroid use without relapses.^6^ In Takayasu arteritis, a systematic review demonstrated that tofacitinib yielded better clinical outcomes than methotrexate, with similar corticosteroid-sparing effects.^7^ Beyond large-vessel vasculitis, JAK inhibitors have shown efficacy in other forms. A case report highlighted tofacitinib’s success in a refractory cutaneous leukocytoclastic vasculitis patient, with complete skin lesion resolution.^8^ Similarly, a retrospective study in polyarteritis nodosa demonstrated tofacitinib’s role in improving skin lesions and achieving disease-free remission, either alone or with corticosteroids.^9^ Of note, regulatory agencies, including the US Food and Drug Administration and European Medicines Agency, have issued boxed warnings for JAK inhibitors because of increased risks—particularly in older adults and those with cardiovascular comorbidities. Observational and pharmacovigilance data further support elevated rates of thromboembolism, stroke, ischemic heart disease, and malignancy in these populations.^10^

Lastly, although current data remain limited to case reports and small case series, the observed efficacy and corticosteroid-sparing effects suggest a promising role for JAK inhibitors in refractory and relapsing disease. Further large-scale, controlled studies are needed to determine their long-term safety, optimal dosing, and positioning within existing treatment paradigms for complex inflammatory and autoimmune diseases.

## Conclusion

This case highlights an incidental but significant remission of GPA following the introduction of baricitinib for alopecia areata universalis. Given the patient’s history of multiple treatment failures and intolerance to traditional immunosuppressive agents, JAK inhibition may represent a promising corticosteroid-sparing therapy for refractory GPA. Further research is warranted to better define their role in these conditions and optimize treatment strategies for patients with overlapping autoimmune diseases.

## Conflicts of interest

None disclosed.
